# Structural basis for the dynamics of human methionyl-tRNA synthetase in multi-tRNA synthetase complexes

**DOI:** 10.1093/nar/gkab453

**Published:** 2021-06-04

**Authors:** Dong Kyu Kim, Hyun Joo Lee, Jiwon Kong, Ha Yeon Cho, Sunghoon Kim, Beom Sik Kang

**Affiliations:** School of Life Sciences, BK21 FOUR KNU Creative BioResearch Group, Kyungpook National University, Daegu 41566, Korea; School of Life Sciences, BK21 FOUR KNU Creative BioResearch Group, Kyungpook National University, Daegu 41566, Korea; Medicinal Bioconvergence Research Center, College of Pharmacy & School of Medicine, Yonsei University, Incheon 21983, Korea; School of Life Sciences, BK21 FOUR KNU Creative BioResearch Group, Kyungpook National University, Daegu 41566, Korea; Medicinal Bioconvergence Research Center, College of Pharmacy & School of Medicine, Yonsei University, Incheon 21983, Korea; School of Life Sciences, BK21 FOUR KNU Creative BioResearch Group, Kyungpook National University, Daegu 41566, Korea

## Abstract

In mammals, eight aminoacyl-tRNA synthetases (AARSs) and three AARS-interacting multifunctional proteins (AIMPs) form a multi-tRNA synthetase complex (MSC). MSC components possess extension peptides for MSC assembly and specific functions. Human cytosolic methionyl-tRNA synthetase (MRS) has appended peptides at both termini of the catalytic main body. The N-terminal extension includes a glutathione transferase (GST) domain responsible for interacting with AIMP3, and a long linker peptide between the GST and catalytic domains. Herein, we determined crystal structures of the human MRS catalytic main body, and the complex of the GST domain and AIMP3. The structures reveal human-specific structural details of the MRS, and provide a dynamic model for MRS at the level of domain orientation. A movement of zinc knuckles inserted in the catalytic domain is required for MRS catalytic activity. Depending on the position of the GST domain relative to the catalytic main body, MRS can either block or present its tRNA binding site. Since MRS is part of a huge MSC, we propose a dynamic switching between two possible MRS conformations; a closed conformation in which the catalytic domain is compactly attached to the MSC, and an open conformation with a free catalytic domain dissociated from other MSC components.

## INTRODUCTION

Aminoacyl-tRNA synthetases (AARSs) catalyse specific aminoacylation of their cognate tRNAs to produce aminoacyl-tRNAs for protein biosynthesis. Among them, methionyl-tRNA synthetase (MRS) ligates a methionine to its cognate tRNAs for both translation initiation and elongation steps during protein synthesis. In eukaryotes, MRS is assembled into a complex with other AARSs. MRS and glutamyl-tRNA synthetase (ERS) form a ternary complex with Arc1p in yeast (1). MRS, ERS, tyrosyl- tRNA synthetase (YRS) and glutaminyl-tRNA synthetase (QRS) form a complex with Tg-p43 in *Toxoplasma gondii* (2). MRS, QRS, and prolyl-tRNA synthetase (PRS) are members of a complex containing three associated proteins in *Trypanosoma brucei* (3). In mammals, nine AARSs including MRS and three ARS-interacting multifunctional proteins (AIMPs) form a multi-tRNA synthetase complex (MSC) (4). Some MSC component proteins utilize additional features such as glutathione transferase (GST)-like domains and leucine zipper motifs to integrate into MSC. Among human MSC components, MRS, glutamyl-prolyl-tRNA synthetase (EPRS), AIMP2, and AIMP3 have a GST domain (5), and specific interactions among these GST domains yield a tetrameric GST complex ordered MRS–AIMP3-EPRS-AIMP2 (6). Further interactions between MSC components enable the assembly of 24 proteins in the case of human MSC, which is estimated to be ∼1.5 MDa and 19 × 16 nm by electron microscopy (EM) (7), and 52 × 14 nm by small-angle X-ray scattering (SAXS) (8). The large discrepancy between the two structural techniques indicates that MSC is conformationally flexible.

Since the structure of MRS from *Escherichia coli* was revealed in 1982 (9), numerous MRS structures have been reported. Two MRS structures from thermophiles have been determined, and their detailed functional mechanisms explored (10,11). Structures of MRS from pathogens, including *Mycobacterium smegmatis* (12), *Leishmania major* (13), *T. brucei* (14) and *Brucella melitensis* (15) have also been revealed, and used to develop specific MRS inhibitors. However, all MRS structures reported to date are from microorganisms, and all share a common catalytic main body structure. Like all members of the class I group of AARS proteins, MRS has a Rossmann fold catalytic domain, a helical anticodon binding domain (ABD), and conserved HIGH and KMSKS motifs that are essential for catalytic activity. MRS also has connecting peptide (CP) and stem contact fold (SCF) domains (16) that are highly conserved from microorganisms to humans. In addition to this catalytic main body, MRSs from some organisms have appendages at the N- and C-termini of the main body (5,17). For example, MRS from *E. coli* has a domain for dimerization at its C-terminus (18), while yeast MRS has a GST domain at its N-terminus that interacts with Arc1P (1). MRSs from *Pyrococcus abyssi* (11) and *Caenorhabditis elegans* (19), respectively, have a dimerization domain and a tRNA binding domain at the C-terminus.

**Figure 1.**
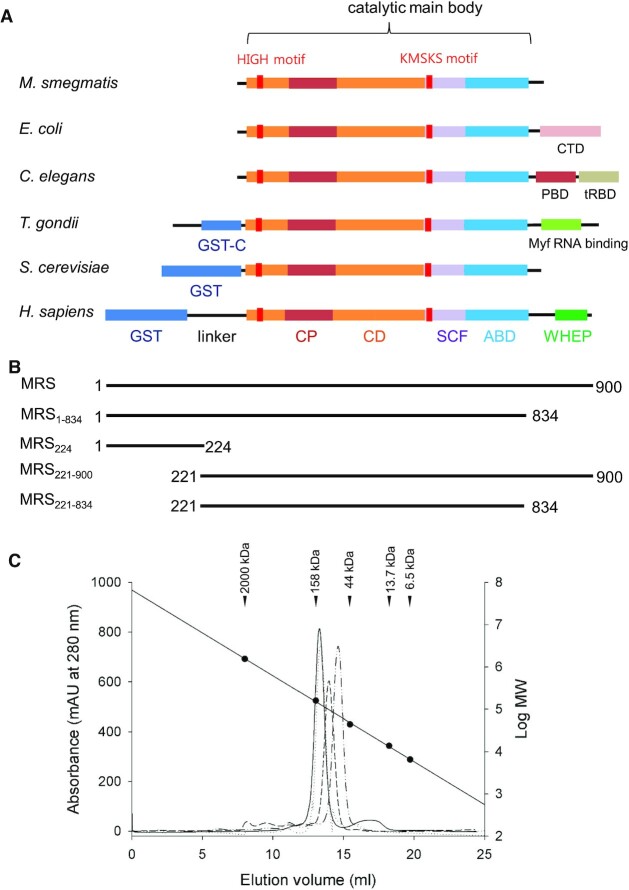
Domain architecture and molecular size of human cytosolic MRS. (**A**) Comparison of the domain architectures of MRSs from various organisms (*Mycobacterium tuberculosis, Escherichia coli, Caenorhabditis elegans, Toxoplasma gondii, Saccharomyces cerevisiae*, and *Homo sapiens*). The catalytic main body consists of a Rossmann fold catalytic domain (CD), connecting peptide (CP), stem contact fold (SCF), and an anticodon binding domain (ABD). There are additional domains at the N- and C-termini of the catalytic main body. Human MRS has a GST domain and a WHEP domain linked by a long linker at the N- and C-terminus, respectively. (**B**) Human MRS constructs used in this study. (**C**) Size estimation by size-exclusion chromatography. The MRS_1–900_–AIMP3 complex (solid), MRS_1–834_–AIMP3 complex (dots), MRS_221–900_ (dashes) and MRS_221–834_ (dots and dashes) were eluted from a Superdex G200 column. Size standards are indicated by black circles.

Human cytosolic MRS is a 900-residue protein, and amino acid sequence analysis indicates additional domains at both termini of human MRS that are not present in MRSs from other organisms (Figure 1A). The conserved main body of MRS consists of a catalytic domain and a helical ABD. At the N- and C-terminus of the main body, human MRS has GST and WHEP domains, respectively. The structure of the C-terminal part (residues 835–900) determined by NMR spectroscopy (https://doi.org/10.2210/pdb2DJV/pdb) revealed a helix-turn-helix motif (K838 to E887) and a lysine-rich peptide region. The lysine-rich cluster and WHEP domain function in tRNA binding and sequestering, respectively (20). At the N-terminus, human MRS has a GST-like domain that interacts with AIMP3, part of the MSC (5). The structure of the GST domain complexed with AIMP3 was determined by X-ray crystallography (6), revealing a domain consisting of GST-N and GST-C subdomains spanning residues M1 to K191. Since the first strand of the Rossmann fold in the catalytic main body is predicted to start at N265, there is a long gap of 73 residues between the GST and catalytic domains. In yeast MRS, the GST domain and catalytic domains are attached almost directly with very few linking residues (21). MRS from *L. major* also has a GST-like domain tightly attached at the N-terminus (13), unlike human MRS.

Because human MSC is a huge assembly of 24 proteins, MRS is a small part of the overall multi-protein complex. MRS in MSC must bind and release tRNA without being hampered by other components to ensure efficient tRNA charging activity, and MRS also needs to interact with other proteins. Under UV irradiation-induced stress, MRS phosphorylation at S662 by GCN2 triggers the release of AIMP3 (22), and S209 and S825 of MRS are phosphorylated by extracellular signal-related kinase (ERK) in response to reactive oxygen species (23). Thus, MRS in MSC is also a substrate of these kinases, and thereby regulates MRS functions. There must be a mechanism by which MRS can be stably assembled into the huge MSC while being fully exposed for easy access by tRNA and proteins such as translational factors and kinases. However, structural information for human MRS remains limited.

To investigate human MRS in MSC, we herein determined crystal structures of the catalytic main body and the N-terminal domain of human MRS, resulting in detailed structures of the catalytic site, tRNA binding site, and the linker region between the GST and catalytic domains. The pronounced flexibility of the linker region suggests a possible conformational change accompanying movement of the catalytic main body relative to the N-terminal GST domain, which attaches to the MSC though AIMP3. We propose that MRS may transition from a closed, compact conformation when attached to the MSC to an open conformation in which MRS is able to bind tRNA for aminoacylation through domain reorientation via the flexible linker peptide.

## MATERIALS AND METHODS

### Cloning, protein expression, and purification

Genes for full-length human MRS (residues 1–900) and AIMP3 were previously cloned into pET30a or pQE80L (24), and the resulting plasmids were used for protein production. The MRS gene was used as a template to generate various deletion derivatives. Mutants (H391A, R399A, R414A and R439A) were generated using a QuikChange Site-Directed Mutagenesis Kit and confirmed by sequencing. To generate a disulfide bond, two cysteines (E385C and S446C) were introduced. An MRS mutant without the zinc knuckles was generated by deleting residues from Q386 to Q445, and including the double mutation E385P/S446G to incorporate a tight turn between β4 and β5 strands. Recombinant plasmids containing genes for truncated MRS (MRS_1–834_, MRS_221–900_, and MRS_221–834_) were transformed into *E. coli* strain BL21 (DE3), and protein expression was induced using isopropyl-β-d-1-thiogalactopyranoside (IPTG) at a final concentration of 0.2 mM. Proteins expressed at 18°C were purified using Ni-NTA affinity and size-exclusion chromatography steps. MRS_1–224_–AIMP3 and MRS–AIMP3 complexes were obtained by co-purification (6), and purified proteins were stored in 20 mM Tris–HCl (pH 7.5) containing 150 mM NaCl.

To estimate the sizes of the MRS variants, purified MRS proteins were subjected to size-exclusion chromatography using a Superdex 200 10/300 column (GE Healthcare) equilibrated in Tris–HCl pH 7.5 containing 150 mM NaCl. The molecular weights of the eluted samples were calculated based on the calibration curve of standard samples.

### Crystallization, diffraction data collection and structure determination

Purified truncated MRS_221–834_ at a concentration of 12.0 mg/ml was crystallized with 21% polyethylene glycol (PEG) 8000 as the precipitant in 0.1 M Tris–HCl (pH 8.0) and 0.2 M MgCl_2_ using the sitting drop vapor diffusion method at 21°C. For diffraction data collection, crystals were soaked in cryoprotectant solution containing 20% (v/v) glycerol and flash-frozen in liquid nitrogen. After a fluorescence scan, X-ray diffraction data were collected at a wavelength corresponding to the zinc absorption peak (1.28273 Å) using synchrotron 5C beam line at the Pohang Accelerator Laboratory (PAL) in Korea. Crystals of the MRS_221–834_ variant belonged to space groups *P*2_1_2_1_2_1_. Another dataset at a resolution of 2.0 Å was collected at a wavelength of 0.9795 Å. Datasets were processed using the HKL2000 program (25). The structure was determined by the single-wavelength anomalous dispersion (SAD) method, and two zinc atoms were identified in the asymmetric unit using Autosol (26). Model building and structure refinement were carried out using COOT (27) and Phenix.Refine (28), respectively. Crystals of the MRS_1–224_–AIMP3 complex were obtained in 20% PEG 8000 and 0.1 M HEPES (pH 7.5). The structure was determined by molecular replacement based on the MRS_1–207_–AIMP3_1–169_ complex using the I3C structure (PDB ID: 4bvx) as template with Phenix.Phaser (29). Model building and structure refinement were carried out using COOT and Phenix.Refine, respectively. Data collection and model statistics are summarized in Table 1.

**Table 1.** Diffraction data collection and refinement statistics

Dataset	MRS(221–834)	MRS(1–224)-AIMP3
Experimental data		
X-ray source	PAL 5C	PAL 5C
Wavelength (Å)	0.9756	0.9795
Space group	*P*2_1_2_1_2_1_	*P*2_1_
Unit cell parameters		
*a*, *b*, *c* (Å)	65.0, 93.0, 122.1	43.2, 71.3, 64.3
Resolution limit (Å)	50–2.0 (2.09–2.02)^a^	50–2.0 (2.07–2.00)
Total reflections	329 227	173 621
Unique reflections	48,954	25,597
Redundancy	6.7 (6.1)	6.8 (5.6)
Completeness (%)	98.2 (94.3)	99.7 (98.8)
*R*_symm_^b^	0.102 (0.624)	0.120 (0.578)
Average I/σ (I)	26.1 (3.7)	25.0 (4.3)
CC_1/2_	0.998 (0.790)	0.994 (0.806)
Refinement details		
Resolutions (Å)	37.3–2.0	39.7–2.0
Reflections (working)	48,889	25,571
Reflections (test)	2000	1292
R_work_/*R_free_*^c^	0.194/0.226	0.189/0.218
Number of waters	345	74
RMSD		
Bond length (Å)	0.002	0.008
Bond angle (°)	0.698	1.280
Average B factors (Å)		
Molecule A (main/side)	39.6 (37.7/41.4)	25.1 (23.5/26.8)
Molecule B (main/side)		22.1 (20.8/23.4)
Waters	43.5	24.0

^a^Numbers in parentheses describe the relevant value for the last resolution shell.

^b^ *R*_sym_ = Σ|*I*_*i*_– |/Σ*I* where *I_i_* is the intensity of the *i*th observation and is the mean intensity of the reflections.

^c^ *R*_work_ = Σ||*F*_obs_| – |*F*_calc_||/Σ|*F*_obs_|, crystallographic *R* factor, and *R*_free_ = Σ ||*F*_obs_| – |*F*_calc_||/ Σ |*F*_obs_| when all reflections belong to a test set of randomly selected data.

### Enzyme assay

MRS aminoacylation activity was assayed as previously described (23) at 37°C with 150 nM proteins in reaction buffer comprising 30 mM HEPES pH 7.4, 100 mM potassium acetate, 10 mM magnesium acetate, 4 mM ATP, 20 μM Met, 1.2 mg/ml tRNAi^Met^, and 25 μCi [^35^S]Met (1000 Ci/mmol; Izotop). Aminoacylation reactions were quenched on 3MM filter paper pre-wetted with 5% trichloroacetic acid containing 1 mM Met. After washing with 5% trichloroacetic acid and drying, radioactivity was detected by a Wallac 1409 liquid scintillation counter (Wallac).

Methionine activation activity of MRS was measured by monitoring the production of pyrophosphate (30). Assays were performed in 20 μl reaction mixture containing 50 mM TRIS-HCl pH 7.5, 100 mM NaCl, 100 mM KCl, 10 mM MgCl_2_, 1 mM dithiothreitol, 250 μM ATP, 0.2 U/ml inorganic pyrophosphatase (Roche), 2.5 mM l-methionine (Sigma-Aldrich) and 1 μM purified MRS–AIMP3 complex or MRS_221–900_ at room temperature. Various concentrations of methionine or ATP were used to obtain *V*_max_ and *K*_M_ values. Reactions were stopped by addition of 180 μl Malachite Green solution (BIOMOL Green; Enzo Life Sciences). After incubation for 25 min, the absorbance at 620 nm was measured using a VesaMax Microplate Reader (Molecular Devices), and the phosphate concentration was calculated from a standard curve generated using various concentrations of inorganic phosphate.

### tRNA pull-down assay

To examine the interactions of MRS_221–900_ and the MRS–AIMP3 complex with tRNA, pull-down assays were performed using Ni-NTA resin. Yeast total tRNA (Sigma-Aldrich) was subjected to a pull-down assay with hexa-His-tagged proteins immobilized on Ni-NTA resin. A 2.5 nmol aliquot of each protein was bound to Ni-NTA resin and washed several times with buffer (50 mM Tris–HCl pH 7.5, 150 mM NaCl). The pull-down assay was performed by adding 1.25 mg yeast tRNA, and resin was washed three times with buffer. The protein and bound tRNA were eluted with buffer containing 200 mM imidazole. UV absorption spectra of the eluted samples and buffers were measured by wavelength scanning from 320 to 230 nm using a UV-1800 UV spectrophotometer (Shimadzu). The amount of tRNA and protein was analyzed by measuring the absorbance at 260 and 280 nm. Synthesized RNA with an elongator tRNA^Met^ sequence labeled with 6-carboxyfluorescein (6-FAM) was purchased from Integrated DNA Technologies and used for the pull-down assay. The amount of RNA eluted was estimated by fluorescence at wavelengths of 495 nm and 517 nm for excitation and emission, respectively.

### Binding affinity measurement by microscale thermophoresis (MST)

MST assays were performed with a Monolith NT.115 instrument (NanoTemper Technologies) (31). Each titration curve consisted of 12 points prepared from a serial dilution of proteins and a constant concentration of 6-FAM-labeled RNA. To measure the binding affinity between MRS and synthetic RNA, 1–100 μM purified MRS_221-900_ or MRS–AIMP3 complex was titrated against 80 nM 6-FAM–RNA. Experiments were performed in phosphate-buffered saline (PBS) and samples were loaded into capillaries. To use unlabeled RNA, proteins were fluorescently labeled using a Monolith His-Tag Labeling kit (Red-tris-NTA Second Generation; NanoTemper Technologies). Purified MRS_221–900_ or MRS–AIMP3 complex (1–100 μM) was titrated against 80 nM 6-FAM–RNA. MST assays were performed with 80% light emitting diode (LED) power using a green filter and 40% MST power. Normalized fluorescence readings (thermophoresis plus T-jump) were plotted as a function of analyte concentration, and curve fitting and dissociation constant *K*_d_ calculation were performed using NanoTemper software.

### Split-luciferase complementation assay

Conformational changes in MRS were monitored by measuring luciferase activity using NanoLuc Binary technology (NanoBit) (Promega). Plasmids for MRS containing Small Bit (SmBit) and AIMP3 fused with SmBit or Large Bit (LgBit) of nanoluciferase were generated previously (32). AARS constructs containing both SmBit and LgBit (LgBit-AARS-SmBit) were built for MRS, aspartyl-tRNA synthetase (DRS), and lysyl-tRNA synthetase (KRS) by insertion of the gene encoding AARS-SmBit into the pBit1.1-N [TK_LgBit] vector (32). Recombinant proteins have linker peptides (GSSGGGGSGGGGSS and SSSGGAQGNSVS for LgBit and SmBit, respectively) between AARS and the nanoluciferase fragments.

CHO-K1 cells (CCL-61, ATCC) were maintained in RPMI-1640 medium (SH30255.01, HyClone) supplemented with 10% (v/v) fetal bovine serum (SH30084.03, HyClone). Plasmids pBiT1.1-N [TK_LgBiT]-MRS-SmBiT and pGEM-T carrying tRNA genes were co-transfected at various ratios, and 48 h after transfection, cell-permeable luciferase substrate (N205A, Promega) was supplemented along with dilution buffer (N206A, Promega) following the manufacturer's instructions, and luminescence was measured by a Glomax 96 microplate luminometer (E4861, Promega).

For L-Met deprivation and addition, 4 μg of the LgBiT-XARS1-SmBiT construct alone was transfected, and transfected cells were seeded as above. At 24 h after transfected cell seeding, L-Met-deprived RPMI-1640 media (cat. LM 011-04, Welgene) or normal RPMI-1640 media (cat. LM 011-01, Welgene) supplemented with 25 mM HEPES (cat. H0887-20ML, Sigma-Aldrich) was combined with L-Met solution (cat. 50272–5ML-F, Merck) and 50 mg/l cycloheximide (cat. 14126, Cayman Chemical Company). A 100 μl volume of the mixture was used to replace the original media and incubated at 37°C for 10 min. Next, a mixture of 1.25 μl of Nano‐Glo live cell substrate and 23.75 μl of Nano‐Glo LCS dilution buffer were added and further incubated for 10 min. Luminescence was subsequently measured as described above.

Sequences of tRNAs used in this study were as follows: initiator tRNA^Met^, 5′-AGCAGAGTGGCGCAGCGGAAGCGTGCTGGGCCCATAACCCAGAGGTCGATGGATCGAAACCATCCTCTGCTA-3′; elongator tRNA^Met^, 5′-GCCTCGTTAGCGCAGTAGGTAGCGCGTCAGTCTCATAATCTGAAGGTCGTGAGTTCGATCCTCACACGGGGCA-3′; tRNA^Lys^(CTT), 5′-GCCCGGCTAGCTCAGTCGGTAGAGCATGAGACTCTTAATCTCAGGGTCGTGGGTTCGAGCCCCACGTTGGGCG-3′; tRNA^Asp^(GTC), 5′-TCCTTGTTAGTATAGTGGTgAGTGTTTCTGCCTGTCATGTGGAGACTGGAGTTTGAGTCCCCAACAGGGAG-3′.

## RESULTS

### Overall structure of the main catalytic body of human MRS

To obtain crystals of human cytosolic MRS for structure determination, we generated a series of MRS variants truncated at the N- and C-termini. Among them, the construct spanning E221 to V834 (MRS_221-834_; Figure 1B) was successfully crystallized. During preparation of the human MRS variants, we estimated their size by size-exclusion chromatography (Figure 1C). As mammalian MRSs digested by trypsin are known to be monomeric proteins (20,33), truncated MRSs without the N-terminal GST domain (MRS_221–900_; 78 kDa) and both N- and C-terminal domains (MRS_221–834_; 70 kDa) were eluted as monomers. Complexes of MRS and AIMP3 (MRS_1–900_-AIMP3; 123 kDa) and MRS without the C-terminal WHEP domain and AIMP3 (MRS_1–834_–AIMP3; 116 kDa) were also eluted as 1:1 heterodimers.

**Figure 2.**
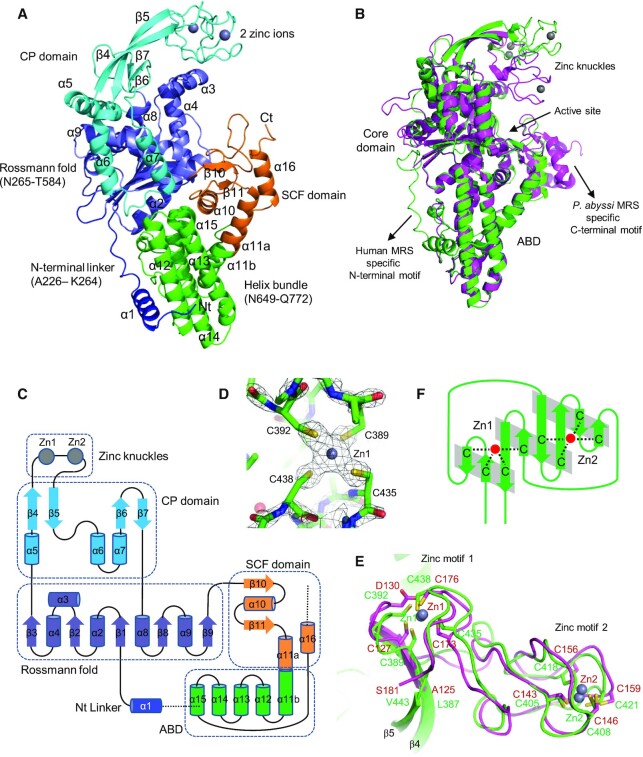
Crystal structures of human cytosolic MRS. (**A**) Ribbon diagram of the catalytic main body of human MRS consisting of a Rossmann fold domain (slate), a CP domain (cyan) containing two zinc atoms (gray spheres), the ABD (green), the SCF (orange), and an N-terminal linker peptide (blue). (**B**) Structural comparison of human MRS (green) and *Pyrococcus abyssi* MRS (magenta). The overall structures superimpose well except for the motifs containing the zinc atoms. (**C**) Topology diagram of MRS. The CP domain containing the zinc knuckles is inserted in the Rossmann fold domain, which is followed by the SCF and ADB domains. (**D**) Electron density map (2*F*_o_ – *F*_c_) at the 3.0 σ contour level showing four sulfur atoms coordinating a zinc atom. (**E**) A roll of eight-stranded β-sheet forms the zinc knuckles. Gray parallelograms indicate hydrogen bonds between main chain atoms. (**F**) Zinc knuckles in human MRS (green) superimposed on those of *P. abyssi* MRS (magenta). Eight cysteines in the zinc knuckles bind the two zinc atoms.

We determined the crystal structure of MRS_821–834_ at a resolution of 2.0 Å (Table 1; Figure 2A). The main catalytic body of MRS without N-terminal GST and C-terminal WHEP domains consists of a Rossmann fold catalytic domain, an ABD helical bundle, and an SCF domain. In addition, the CP domain inserted in the Rossmann fold and a peptide extension (A226–R264) at the N-terminus of the catalytic domain were identified. The overall structure of human MRS is most similar to that of *P. abyssi* MRS (11) among the MRS structures determined to date (Figure 2B). The root mean square deviation (RMSD) between the C_α_ atoms of human and *P. abyssi* MRS structures was 0.428 Å according to the DALI server (34).

In the crystal structure, residues N265–K593 form a Rossmann fold with a five-stranded β-sheet ordered β3–β2–β1–β8–β9 (Figure 2C). The CP domain is inserted in the loop connecting strand β3 and helix α8 preceding strand β8 in the Rossmann fold. The CP domain consists of three helices (α5, α6 and α7) surrounding one side (α8 and α9) of the Rossmann fold, a long twisted β-hairpin structure (β4 and β5) inserted between α5 and α6 helices, and two compact knuckle structures, which are introduced in the hairpin loop. X-ray absorption scanning of MRS crystals revealed a zinc absorption peak, allowing structure determination via Zn-based single-wavelength anomalous dispersion (SAD). Two zinc atoms are present in the knuckle structures, each ligated by four cysteines (C389, C392, C435, and C438 for Zn1; C405, C408, C418 and C421 for Zn2; Figure 2D and E). The topological fold of the zinc-containing domain is a roll formed from a β-sheet containing eight strands (Figure 2F). This fold is highly conserved with that in *P. abyssi* MRS, which employs an aspartate and seven cysteines for zinc coordination. This compact domain containing two zinc atoms is also similar to the domain in *E. coli* MRS (16) harboring one zinc atom. Helix α11 is kinked due to N648, which bulges out of the helix. The N- and C-terminal parts of the helix (α11a and α11b) participate in the SCF and ABD, respectively. The ABD is a bundle of five helices (α11B to α15). A long peptide following helix α15 traverses back toward the SCF domain, and allows helix α16 to be located near helix α11a to complete the SCF domain (Figure 2A and C).

### Substrate binding sites

**Figure 3.**
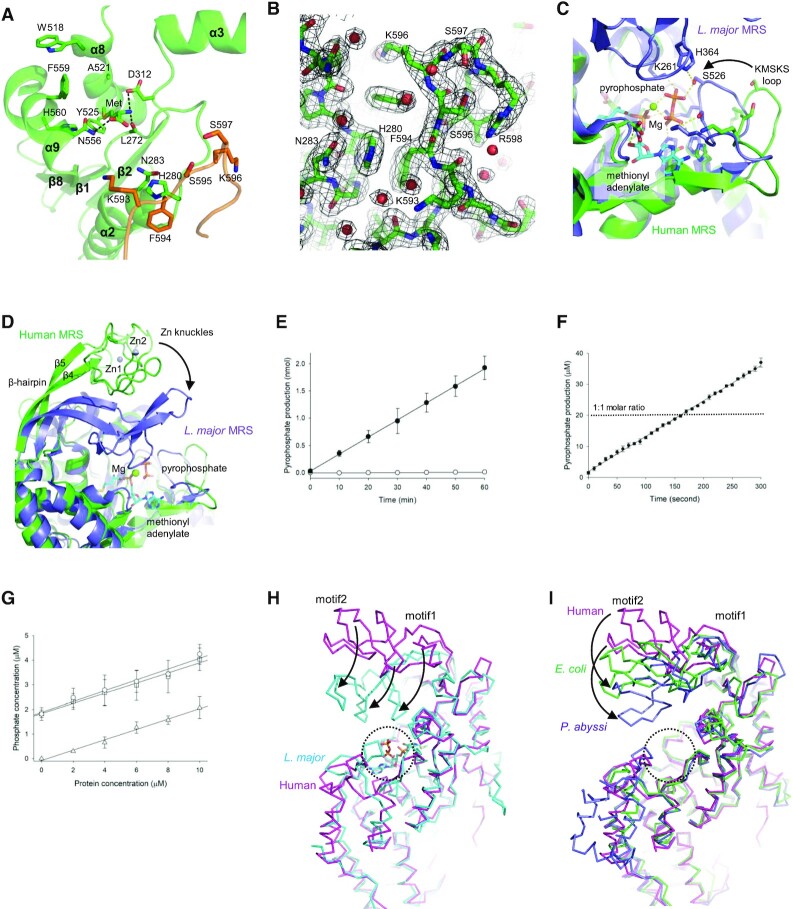
Catalytic site in human MRS. (**A**) A deep pocket for methionine and ATP in the Rossmann fold domain is surrounded by helices α3, α8 and α9, and the KFSKS loop (orange). A methionine fits snugly in the pocket, and L272, D312 and N556 can interact with the amino and carboxyl groups of methionine. N283 and H280 form a floor for ATP along with the conserved HIGH motif of MRS. (**B**) The positions of the residues in the HIGN motif and the KFSKS loop are well-defined by the electron density map (2*F*_o_ – *F*_c_) at the 1.0 σ contour level. (**C**) Superimposition of the active site of human MRS (green) on that of *Leishmania major* MRS (slate) containing methionyl adenylate and pyrophosphate (sticks). The KFSKS loop of human MRS is predicted to move in order to interact with reaction intermediates and substrates, as shown for *L. major* MRS. K261 and H364 in a motif corresponding to the zinc knuckles are involved in interactions with the pyrophosphate. (**D**) The zinc knuckles of human MRS are predicted to move by bending the β-hairpin structure (β4–β5) in the CP domain. (**E**) Pyrophosphate production by the methionine activation reaction of full-length MRS (closed circles) and truncated MRS without zinc knuckles (open circles). (**F**) No burst production of pyrophosphate was observed in methionine activation reaction of MRS. Pyrophosphate production by 20 μM MRS was measured in the presence of inorganic pyrophosphatase. The concentration of pyrophosphate was linearly increased. A single turnover of MRS (production of 20 μM pyrophosphate) was achieved after about 160 seconds. (**G**) Methionine activation reactions were performed with various concentrations (0, 2, 4, 6 and 10 μM) of the MRS mutant without zinc knuckles for 5 min in the presence of inorganic pyrophosphatase. Phosphate production from methionine activation reaction (open circles) was not observed. Small amount of phosphate was also detected in the absence of ATP (open triangles) or methionine (open rectangles). (**H**) Superimposition of the structure of human MRS (magenta) on that of *L. major* MRS (cyan). Arrows indicate the corresponding positions, which can be achieved by bending and twisting the β-hairpin structure. Motif1 reaches the active site (dotted circle). (**I**) Superimposition of the structure of human MRS (magenta) on those of *E. coli* MRS (green) and *P. abyssi* MRS (blue). Arrows indicate movements of motifs by bending the β-hairpin structure. Motif2 reaches the active site (dotted circle).

The catalytic site in MRS is located at a crevice at the C-terminal ends of the Rossmann fold β-strands (β1, β2, and β8). A large cavity for the binding of methionine and ATP is present at the crevice, with helices α3, α8, and α9, and the ^593^KFSKS^597^ loop (corresponding to the KMSKS motif in *E. coli* MRS) surrounding the cavity (Figure 3A and B). When a methionine was placed at the cavity, residues for positioning a methionine are appropriately located, consistent with the *E. coli* MRS-methionine complex structure (35). At the end of strand β1, H280 and N283 from the ^280^HLGN^283^ motif (corresponding to the HIGH motif in *E. coli* MRS) that serves as a floor for ATP.

When the structure of human MRS is superimposed on the *P. abyssi* MRS structures, the Rossmann fold is well-conserved (see Figure 2B). However, there is a discrepancy regarding the positions of the zinc knuckles. The RMSD between the two structures decreased to 0.292 Å without the zinc knuckles (C389 to C441 for human MRS and C127 to I180 for *P. abyssi* MRS), and the RMSD between two zinc knuckle structures was also 0.292 Å. In the human MRS structure, the zinc knuckles are located far from the methionine binding site, and the catalytic site is spacious enough to accommodate a methionine and an ATP molecule. For structural analysis of possible movements of the motifs at the catalytic site, the human MRS structure was superimposed on the structure of *L. major* MRS complexed with methionyl adenylate and pyrophosphate (13) (Figure 3C and D). The Rossmann fold structure and the HLGN motif (HIGH motif in *L. major* MRS), which serve as a floor for methionyl adenylate and pyrophosphate, superimpose well. Dramatic conformational changes are expected for the two motifs upon interaction with pyrophosphate. The KFSKS loop (KISKS loop in *L. major* MRS) folds into the ATP binding site to allow the lysine and serine residues to form hydrogen bonds with the pyrophosphate, and the Zn knuckles move close to the ATP binding site to cover the methionyl adenylate and pyrophosphate.

### Movement of the zinc knuckles

In the case of *L. major* MRS, the motif corresponding to the zinc knuckles provides residues that hold pyrophosphate during the methionine activation reaction. To investigate whether the zinc knuckles are essential for the catalytic activity of human MRS, we generated a truncation mutant lacking the zinc knuckles, and monitored pyrophosphate production (Figure 3E). In the presence of pyrophosphatase, human MRS continued to generate pyrophosphate, and inorganic phosphates accumulated. However, pyrophosphate production did not occur with the MRS mutant lacking the zinc knuckles. This could be due to a failure in enzyme turnover. Phosphate production was monitored in the early stage of the reaction with 20 μM MRS (Figure 3F). No burst production of pyrophosphate was observed in the methionine activation reaction. It implies that methionyl adenylate does not stay in the active site without pyrophosphate. We also monitored the phosphate concentration after 5 min of reaction with various concentrations of the MRS mutant. The mutant was unable to produce pyrophosphate even in the first round of reaction (Figure 3G). Therefore, the zinc knuckles are required for the synthesis of methionyl adenylate, and the absence of the zinc knuckles does not stabilize methionyl adenylate.

Since the zinc knuckles are located far from the ATP binding site in the human MRS structure, the β-hairpin structure holding the knuckles should be bent so that the knuckles approach the active site cavity, as observed in the *L. major* MRS structure (Figure 3D). Because *L. major* MRS does not have a motif corresponding to the second zinc motif (motif2), the first zinc motif (motif1) in human MRS is brought close to the active site by bending and twisting the β-hairpin structure (Figure 3H). We also superimposed the human MRS structure on the structures of *E. coli* and *P. abyssi* MRSs. The zinc knuckles of *P. abyssi* MRS are positioned close to the active site, while those of *E. coli* MRS are located midway between those in human and *P. abyssi* MRSs (Figure 3I). This type of bending introduces motif2 into the active site.

**Figure 4.**
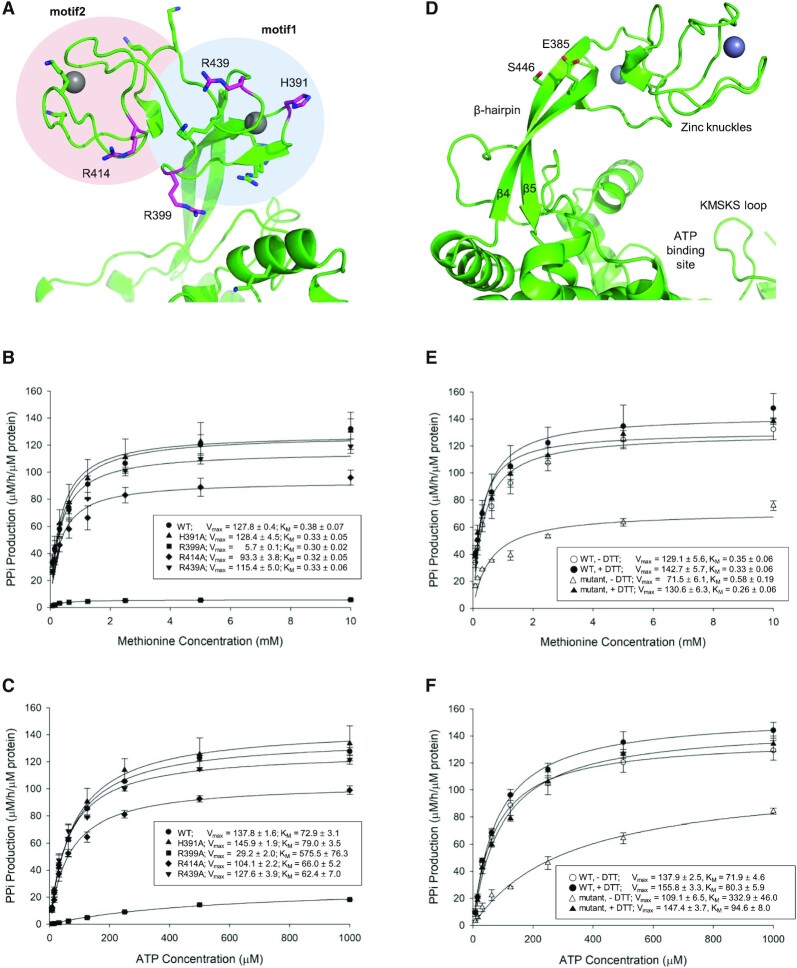
Methionine activation activities of MRS and its mutants. (**A**) Residues in the zinc knuckles mutated to alanine. H391, R399, and R439 are positioned in motif1, while R414 is located in motif2. Other positively-charged residues (sticks) cannot approach the active site. Pyrophosphate production rates of MRS and its mutants in the presence of inorganic pyrophosphatase were measured under various concentrations of methionine (**B**) and ATP (**C**). The R399A mutant (triangles) lost the most the activity. The activities of H391 (inverted triangles), R414 (squares), and R439 (diamonds) mutants are comparable to that of the wild type enzyme (circles). (**D**) E385 in β4 and S446 in β5 were mutated to cysteine. The positions of these residues are suitable to engage in disulfide bonds. Pyrophosphate production rates of MRS and the E385C/S446C double mutant were measured in the presence of inorganic pyrophosphatase under various concentrations of methionine (**E**) and ATP (**F**). The double mutant lost its activity (solid triangles) and the activity was recovered in the presence of the reducing agent dithiothreitol (open triangles). The activity of the wild type without dithiothreitol (open circles) is similar to that with the reducing agent (closed circles). *V*_max_ and *K*_M_ values are shown in the insert (*n* = 4; mean ± SD).

To establish which residue is responsible for holding ATP during the catalytic reaction, four positively-charged residues that can potentially access the ATP binding site by bending and twisting the β-hairpin structure were mutated into alanine: H391, R399 and R439 located at motif1, and R414 at motif2 (Figure 4A). When the pyrophosphate production activities of the mutants were measured, the R399A mutant exhibited a dramatic loss in activity (Figure 4B and C). Because we measured the activities at various concentrations of methionine and ATP, we could compare the effects on interaction with methionine and ATP separately. Although the K_M_ value of methionine for R399 (0.30 μM) is similar to the values obtained for the wild-type enzyme and other mutants (0.32–0.38 μM), the *K*_M_ value of ATP for R399 (575.5 μM) is much greater than for the others (62.4–79.0 μM). These results suggest that the side chain of R399 plays a role in holding ATP. Although the R414A mutant exhibited a slight decrease in activity, the double mutant (R399A/R414A) achieved similar levels of pyrophosphate production to that of the R399A mutant (data not shown).

To confirm that the bending of the β-hairpin structure is essential for catalytic activity, we introduced a disulfide bond to restrict the motion of the β-hairpin by introducing the double mutation E385C/S446C (Figure 4D). This mutant displayed a dramatic loss of pyrophosphate production activity (Figure 4E and F). However, its activity was recovered in the presence of the reducing agent dithiothreitol. The activity of wild type MRS was not affected by the reducing agent. The *K*_M_ values of ATP for mutant and wild-type enzymes are 322.9 and 71.9 μM, respectively, and those of methionine for the mutant and wild-type enzymes are 0.58 μM and 0.35 μM, respectively. Again, the increase in the *K*_M_ for ATP is much greater than that for methionine.

### The tRNA binding site

**Figure 5.**
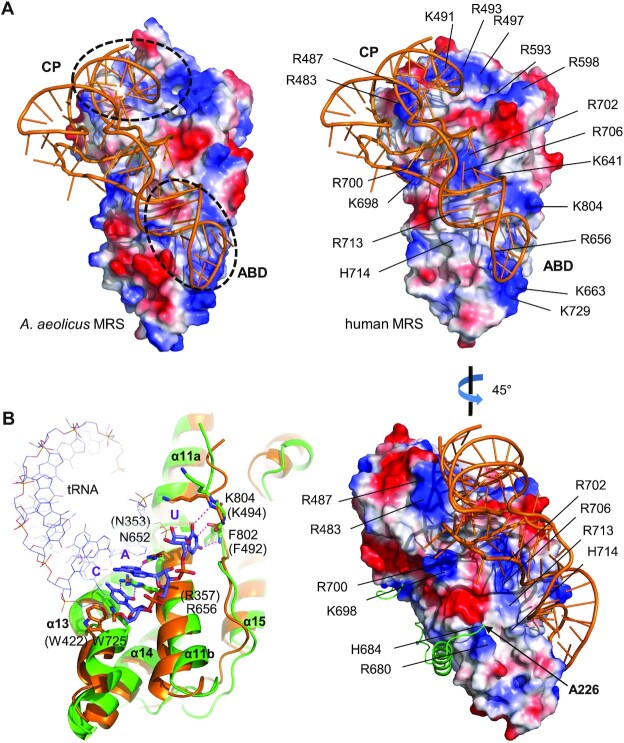
Structure of the tRNA binding site. (**A**) Comparison of the surface potential of tRNA binding sites. tRNA (orange) is placed on human MRS (right) as observed in the *Aquifex aeolicus* MRS–tRNA complex (left). There are two major binding sites for tRNA in MRS, and both are negatively-charged areas at the CP and ABD (indicated by dashed circles). Positively- and negatively-charged areas are colored blue and red, respectively. The N-terminal end (A226) faces the tRNA (right bottom). (**B**) tRNA anticodon binding site. Human MRS (green) is superimposed on *A. aeolicus* MRS (orange). tRNA is shown in wire representation. The anticodon (C, A, and U) and residues interacting with the anticodon are shown as sticks.

The crystal structure of MRS bound to tRNA was determined for MRS from *Aquifex aeolicus* (36). Superimposition of the human MRS structure on that of the *A. aeolicus* MRS–tRNA complex allowed us to predict the tRNA binding mode in human MRS (Figure 5). Two major points of contact in MRS are the CP domain (for interaction with the acceptor arm) and the ABD (for binding to the anticodon stem and loop). Surface electrostatic potential analysis revealed that the pattern of positively-charged regions on the surface of human MRS is similar to that of *A. aeolicus* MRS, where tRNA contacts.

The arrangement of helices in the human MRS ABD is structurally similar to that in *A. aeolicus* MRS (Figure 5B). The sugar-phosphate backbone of the tRNA anticodon stem and loop lies on the surface of helices α11 and α13. Determining tRNA specificity, three nucleotides of the anticodon contact residues in helix α11, the helical turn following helix α13, and the peptide extended to helix α16. Residues N353, R357, W422, F492 and K494 in human MRS correspond to N652, R656, W725, F802 and K804 that interact with the anticodon nucleotides C, A and U in *A. aeolicus* MRS, and all are conserved. We placed the tRNA molecule in the *A. aeolicus* MRS-tRNA complex on the human MRS structure (Figure 5B). The indole ring of W725 stacks against the pyrimidine ring of the cytosine, which forms two hydrogen bonds with the guanidino group of R656. Additionally, N652 forms a hydrogen bond with the adenine, while the uracil moiety engages in three hydrogen bonds with the main chain amide and carbonyl oxygen of the conserved F802 and K804 residues.

### Structure of the N-terminal linker peptide

As shown in Figure 1, human MRS has a long linker peptide between the N-terminal GST domain and the catalytic Rossmann fold domain. The crystal structure of MRS_221–834_ revealed the conformation of the C-terminal part of the linker (A226–R264) connected to the catalytic domain. The structure of the first five residues in the protein could not be determined due to poor electron density, suggesting it is flexible.

**Figure 6.**
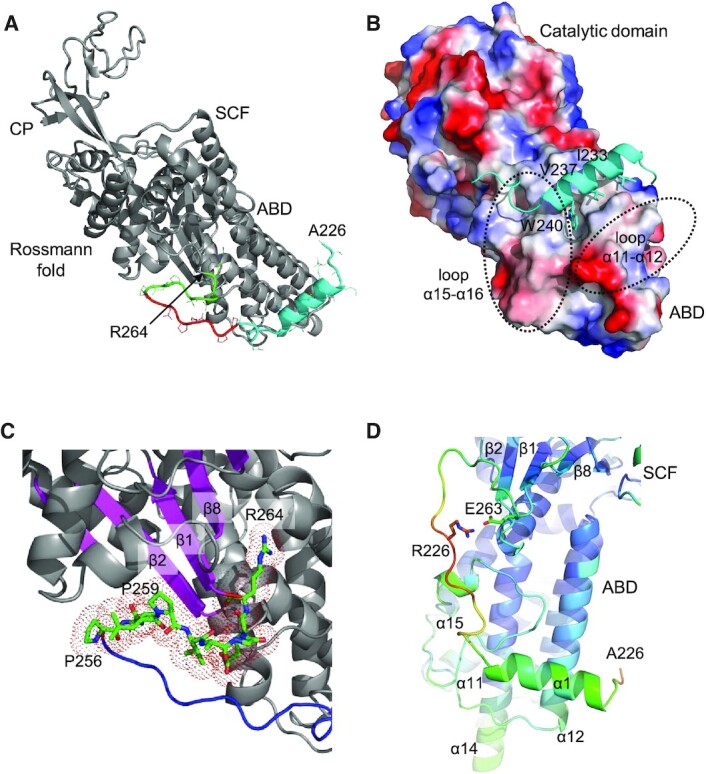
Structure of the N-terminal linker peptide. (**A**) Ribbon diagram of MRS_221–834_. The N-terminal linker peptide, from A226 to R264 (colored cyan, red, and green), wraps around the ABD. (**B**) The first α-helix at the N-terminus of the peptide is attached to the ABD. The side chains of I233, V237 and W240 are inserted in a hydrophobic groove in the ABD. Two loop regions (α11–α12 and α15–α16) holding the helix are indicated by dotted circles. Electrostatic surface potential is colored as in Figure 5. (**C**) The C-terminal region (P256 to R264; shown as sticks with dots) of the peptide surrounds the N-terminal ends of the β-strands (magenta) in the catalytic Rossmann fold domain. (**D**) The central region of the peptide is not attached firmly to the catalytic domain. This region has higher B-factors (red) compared with the ABD (blue indicates a low B-factor).

The structure comprises three segments: the first α-helix, an extended peptide, and a peptide attached to the Rossmann fold domain (Figure 6A). The first N-terminal helix (E230–K242) interacts with the ABD through hydrophobic interactions. The hydrophobic surface of the helix, which includes I233, A236, V237 and W240, binds to the hydrophobic patch on the ABD composed of M672, L674, L682, A683, L744 and F785. The binding area covers ∼800 A^2^. The indole side chain of W240 is embedded in a distinct pocket in the ABD (Figure 6B). The helix is perpendicular to helices in the ABD, and located between two loops connecting helices α11–α12 and α15–α16.

Near the Rossmann fold domain, residues P256–R264 lie atop the N-terminal ends of the strands in the β-sheet (Figure 6C). This part of the structure is known to be essential for MRS catalytic activity (37). However, it is unlikely to participate in the catalytic reaction directly because the active site is at the C-terminal end of the β-sheet strands, while the tRNA binding site is located far from this region. Rather, it appears to contribute to catalytic activity by stabilizing the Rossmann fold domain, and thereby maintaining the correct active site conformation. In this region, hydrophobic residues P256, V257, L258 and P259 fill the gap between the β-sheet and the helical layer of the Rossmann fold through interactions with Y532, Q535 and W539. The side chain of R264 forms another cap at the opposite side of the β-sheet through hydrogen bonds to D547 and Y549. These strands (β1, β2 and β8) and helices (α2 and α8) contacting this peptide region are important for forming the catalytic site.

Unexpectedly, the central part of the N-terminal peptide region (G243–N255) is separate from the main catalytic body of MRS, unlike the N- and C-terminal parts of the peptide region, which are directly attached to the ABD and the catalytic Rossmann fold domain, respectively. Residues in this peptide region have relatively high B-factors and do not participate in interactions with other MRS regions, apart from an ionic interaction between R251 and E263 (Figure 6D).

### A structural model for full-length MRS

**Figure 7.**
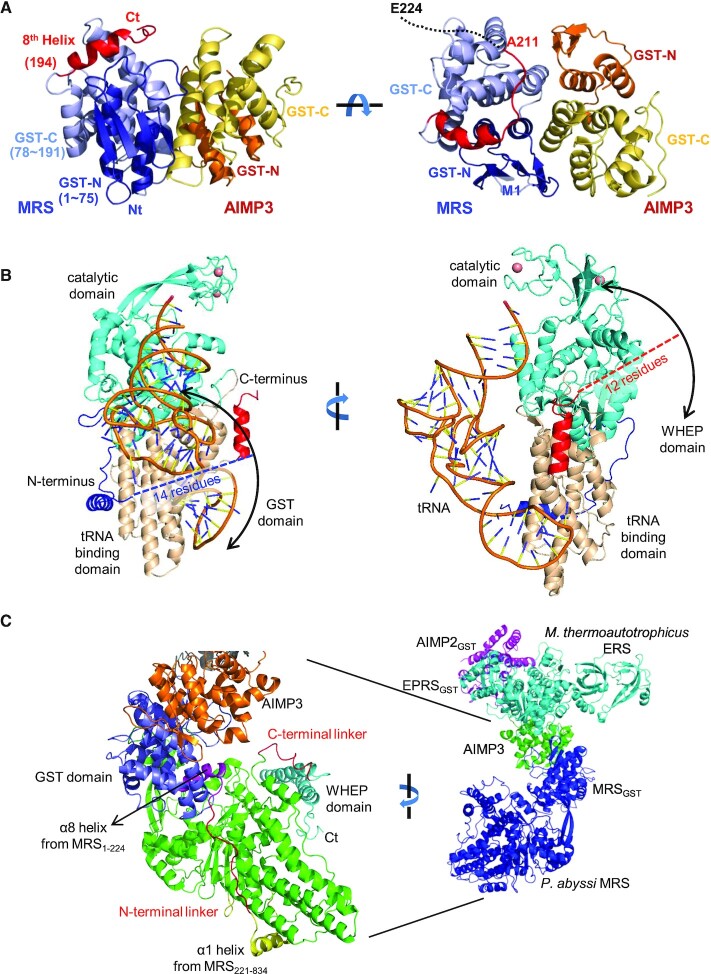
Model of full-length MRS. (**A**) Ribbon diagram of the MRS_1–224_–AIMP3 complex. Helix α8 (red) is positioned between the GST-N (blue) and GST-C (light blue) domains of MRS, and the C-terminal peptide envelops GST-C. (**B**) Possible locational ranges of the N-terminal GST and C-terminal WHEP domains. The tRNA bound to the MRS is represented by wire and sticks, as in Figure 5. The N-terminal linker peptide and the GST domain may remain in the position of the tRNA. (**C**) Model of full-length MRS complexed with AIMP3 (left) based on the previous EM structure (right). The N-terminal linker peptide traverse the tRNA binding surface.

A heterodimeric complex structure of GST domains from MRS and AIMP3 was determined in previous work (6) using an MRS construct spanning residues M1–Q207. To determine the structure of the region between the MRS_1–207_ and MRS_221–834_ constructs, we generated an extended version of the MRS GST domain (MRS_1–224_) and determined the crystal structure of the GST domain complexed with AIMP3 at a resolution of 2.0 Å (Table 1). Although the structure of the MRS_1–224_-AIMP3 complex is almost identical to the previous structure, we were able to determine the position of a few additional residues (Figure 7A). The GST domain in human MRS includes the eighth helix that corresponds to the ninth helix of the theta isoform of human GST (38). Helix α8 is kinked due to the presence of P200, and it lies above the crevice between the GST-N and GST-C subdomains of MRS. The peptide next to this helix is visible up to A211, and it wraps the GST-C subdomain. Electron density for the C-terminus (E212–E224) is poor, implying that it is flexible and separate from the GST domain.

Structures of the MRS catalytic main body (A226–A822) and two additional domains at the N- and C-termini (M1–A211 and T835–K900, respectively) have been determined, and two peptide regions are missing; 14 residues (^212^EGRAVTNEPEEEEL^225^) after the N-terminal GST domain, and 12 residues (^823^KTSPKPAVVETV^834^) before the C-terminal WHEP domain. These peptides must be flexible since they are not visible in the electron density in the crystal structures of MRS_1–224_ and MRS_221–834_. Considering the lengths of the missing segments, the N- and C-terminal domains must be less than 40 Å and 35 Å away from the main body, respectively (Figure 7B). The positions of the N- and C-terminal domains can be roughly predicted based on the directions of the visible ends at the main body.

Since MRS is attached to MSC though the N-terminal GST domain, we investigated the relative positions of the GST domain and the catalytic domain, which depend on the location of the long flexible N-terminal linker peptide. Notably, the N-terminal peptide of MRS_821–834_ (A226) is oriented toward the tRNA binding site (see Figure 5A). The carbonyl group of A226 is hydrogen bonded to the side chains of R680 and H684 in the ABD, and near to the amino group of A226 are positively-charged residues R702, R706, R713 and H714 on the surface of the ABD. These residues could accommodate the cluster of glutamic acid residues (^219^EPEEEE^224^) present in the N-terminal missing peptide, and the N-terminal GST domain may be positioned near the ABD.

Because the missing peptide is flexible and relatively long, the GST domain of MRS (MRS_GST_) could be located far from the catalytic main body. However, a previous electron microscopy (EM) study on the MRS–AIMP3–EPRS_GST-ERS_–AIMP2_GST_ complex suggested a model in which the MRS catalytic domain is located near the tetrameric complex of GST domains (6). In the EM model, MRS is compact and the catalytic main body is located close to the MRS_GST_ (Figure 7C).

The observed C-terminal end (A822) of the main body is located near the KFSKS loop, and the C-terminal WHEP domain is not far from this loop. The C-terminal WHEP domain plays a role in the efficient capture of tRNA (20). Deletion of the WHEP domain dramatically increases the dissociation constant (K_d_) of MRS for the acceptor stem of tRNA^Met^. Thus, the WHEP domain is predicted to be located near the tRNA acceptor stem in MRS-tRNA complexes, meaning that both N- and C-terminal domains of MRS would face the tRNA binding surface of the ABD (Figure 7C).

### Human MRS adopts two conformational states

**Figure 8.**
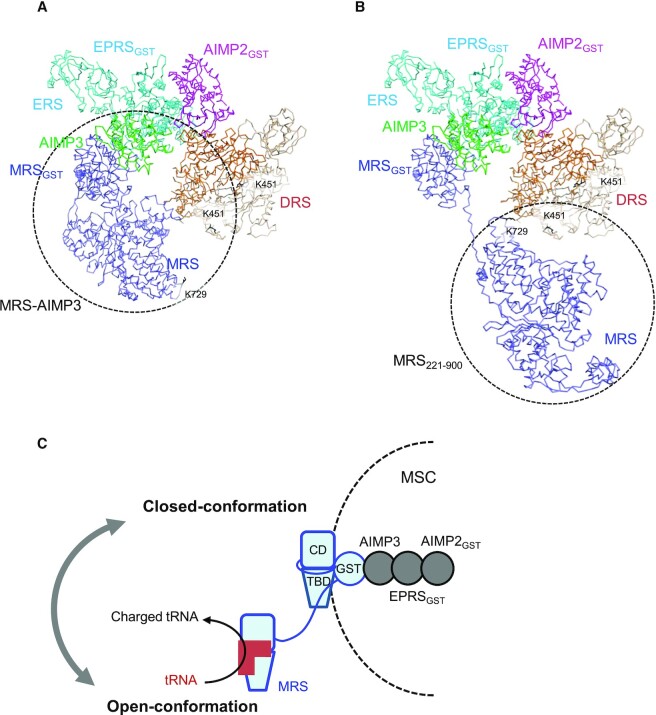
Schematic diagram of the conformational dynamics of MRS. (**A**) Model of the closed conformation of MRS. The catalytic domain of MRS is closely attached to other MSC components. The MRS_GST_–AIMP3-EPRS_GST_–AIMP2_GST_-DRS complex is modeled as described previously (24). Dashed circles indicate the MRS–AIMP3 complex, which was used for the enzyme assay. The side chains of K729 of MRS and K451 of DRS are shown as black sticks. (**B**) Model of the open conformation of MRS. The catalytic domain of MRS is released from other MSC components. Dashed circles indicate MRS_221-900_, which was used for the enzyme assay. (**C**) MRS in the MSC exists in an equilibrium state between open and closed conformations.

In the above model of a full-length MRS, the N-terminal linker peptide of MRS envelops the tRNA binding site. The MRS catalytic main body is closely associated with MRS_GST_ and other MSC components in the EM model of MRS complexed with AIMP3, EPRS_GST-ERS_ and AIMP2_GST_ (Figure 8A). In this conformation, MRS is relatively compact, and it is difficult for tRNA^Met^ to bind to the tRNA binding site because it is blocked by the GST domain and the N-terminal linker peptide. This closed conformation will therefore be inactive for tRNA charging. Active MRS must be in an open conformation, in which the linker peptide is released from the tRNA binding site and the catalytic main body is apart from its GST domain and other MSC components to allow tRNA^Met^ to easily access the binding site (Figure 8B).

Because AIMP3 directly contacts DRS and MRS_GST_ is located close to DRS (24), the catalytic domain of MRS can be positioned near DRS. The 3-dimensional architecture of MSC was recently determined using cross-linking mass spectrometry (39). This work revealed a cross-link between K451 of DRS and K729 of MRS. K729 is at the opposite end of the ABD to the zinc knuckles in MRS, and it must approach close to DRS to form the cross-link. The distances from the end (A211) of MRS_GST_ to two K451 residues in a DRS dimer are 59 and 62 Å in the MRS–AIMP3–EPRS–AIMP2-DRS model. The space between A221 and K729 in the MRS_221–834_ structure is 31 Å. Thus, the N-terminal linker in MRS must be flexible in the open conformation of MRS to bridge the gap and allow cross-linking.

**Figure 9.**
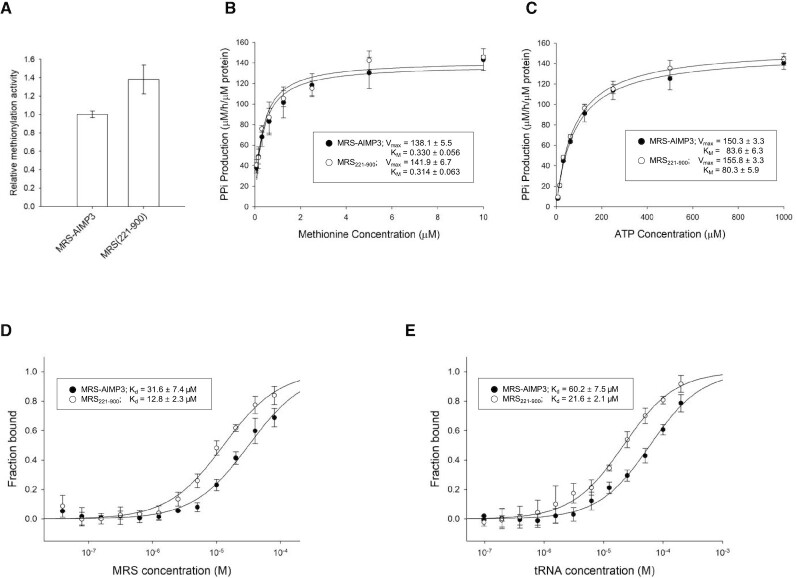
Activities of the MRS–AIMP3 complex and MRS without its N-terminal GST domain. The activities for methionylation of tRNA (**A**) and pyrophosphate production by methionine activation reaction in various concentrations of methionine (**B**) and ATP (**C**) are shown for the MRS–AIMP3 complex and MRS_221–900_. *V*_max_ and *K*_M_ values are shown in the insert (*n* = 4; mean ± SD). The tRNA binding affinities of the MRS–AIMP3 complex and MRS_221–900_ toward 6-FAM-labeled synthetic RNA^Met^ (**D**) and those of synthetic RNA^Met^ toward the fluorescently-labeled MRS–AIMP3 complex and MRS_221–900_ (**E**) were measured by microscale thermophoresis (MST) experiments. *K*_d_ values are shown in the insert (*n* = 4; mean ± SD).

MRS appears to reside in the closed conformation based on the EM structure. However, conversion of MRS to the open conformation is necessary for aminoacylation of tRNA^Met^ (Figure 8C). The two conformational states of MRS must therefore coexist. In the MRS–AIMP3 complex, MRS is likely present in a dynamic equilibrium between open and closed conformations. MRS_221–900_, which lacks the N-terminal GST domain, must only adopt the open conformation. We compared the catalytic activities of the MRS–AIMP3 complex and MRS_221–900_, and the tRNA charging activity of MRS_221–900_ was ∼40% higher than that of the MRS–AIMP3 complex (Figure 9A).

AARS ligates an amino acid to its cognate tRNA in two steps: amino acid activation and tRNA charging. During the first step, an amino acid and an ATP molecule bind at the catalytic active site and react to yield aminoacyl-AMP and pyrophosphate products. In the closed conformation, the N-terminal GST domain of MRS may block the catalytic site. To determine whether the higher activity of MRS_221–900_ is due to the availability of the tRNA binding site or the catalytic site for methionine and ATP, we compared the amino acid activation activities of the MRS–AIMP3 complex and MRS_221–900_. Pyrophosphate production resulting from the methionine activation reaction was monitored by measuring phosphate in the presence of inorganic pyrophosphatase (Figure 9B and C). *V*_max_ and *K*_M_ values for methionine with MRS–AIMP3 at 0.25 mM ATP concentration were 138.1 μM/h/μM protein and 0.330 μM, respectively. These values are comparable to those of MRS_221–900_ (141.9 μM/h/μM protein and 0.314 μM, respectively). With 2.5 mM methionine, *V*_max_ and *K*_M_ values for ATP with MRS–AIMP3 were 150.3 μM/h/μM protein and 83.6 μM, respectively, while for MRS_221–900_ these were 155.8 μM/h/μM protein and 80.3 μM, respectively.

Thus, the superior tRNA charging activity of the truncated MRS_221–900_ could be due to greater tRNA binding ability. To investigate this, hexa-His-tagged MRS proteins were pulled down using yeast total tRNA and Ni-NTA resin. The amount of tRNA eluted with MRS was estimated from the increase in absorbance at 260 nm. Interestingly, the amount of tRNA associated with MRS_221–900_ was almost twice that with the MRS–AIMP3 complex, and the *A*_260_/*A*_280_ ratio indicated an increase due to nucleic acid (data not shown). Absence of the linker peptide and the GST domain around the tRNA binding site therefore enhances the binding of tRNA. Without the GST domain, MRS may bind other tRNA species, resulting in an increase in total tRNA interacting with MRS. Indeed, MRS can bind tRNA^Lys^ under oxidative stress conditions, resulting in misacylation (23), and this may be because absence of the GST domain relaxes the tRNA binding specificity of MRS. To exclude this possibility, we measured the fluorescence intensity of synthetic tRNA^Met^ labeled with 6-FAM after pull-down with hexa-His-tagged MRS proteins. Again, truncated MRS_221MRS–AIMP900_ displayed greater binding to tRNA^Met^ than the MRS–AIMP3 complex. The fluorescence intensity of MRS_221MRS–AIMP900_ was increased almost 2-fold with the RNA, compared with the MRS–AIMP3 complex (data not shown).

The binding affinities of MRS_221MRS–AIMP900_ and the MRS–AIMP3 complex for the synthetic RNA were measured by MST assay, and *K*_d_ values for the MRS–AIMP3 complex and MRS_221–900_ were 31.6 μM and 12.8 μM, respectively (Figure 9D). To exclude the possible effect of the fluorophore in the tRNA on the interaction between protein and RNA, the MST titration assay was repeated with fluorescently-labeled protein. As expected, the affinity of MRS_221–900_ (*K*_d_ = 21.6 μM) for the RNA without 6-FAM was greater than that of the MRS–AIMP3 complex (*K*_d_ = 60.2 μM; Figure 9E).

### Dynamics of MRS *in vivo*

**Figure 10.**
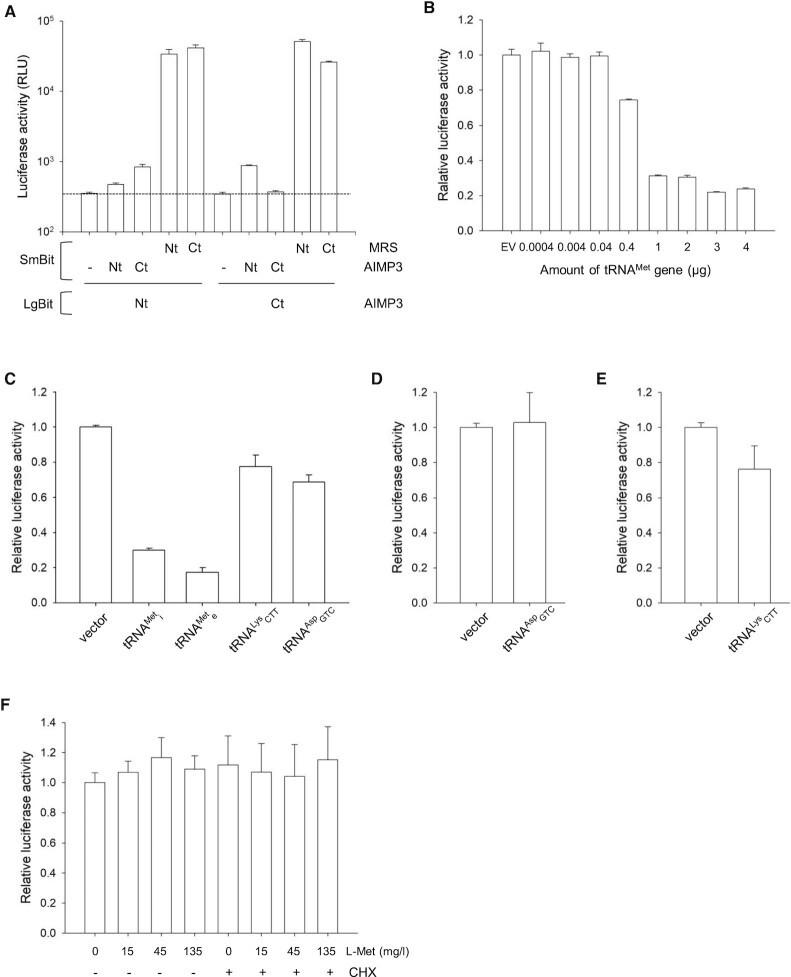
Conformational changes of MRS *in vivo*. (**A**) Both the N- and C-termini of MRS are spatially close to AIMP3. Large Bit (LgBit) connected to AIMP3 and Small Bit (SmBit) linked to MRS were co-expressed in CHO-K1 cells and combined to measure luciferase activity. The activity with SmBit at the N-terminus of MRS is comparable to that with SmBit at the C-terminus of MRS, with LgBit at the N- and C-terminus of AIMP3. The proximity of the MRS C-terminus to AIMP3 is comparable to that of the MRS N-terminus, which binds AIMP3. (**B**) MRS containing both LgBit and SmBit at the N- and C-termini (LgBit-MRS-SmBit). The luciferase activity of LgBit-MRS-SmBit in a closed conformation, in which the N- and C-termini of MRS are closely located, was reduced by increasing tRNA^Met^ expression. (**C**) The luciferase activity of LgBit-MRS-SmBit was measured in the presence of various tRNA genes. Luciferase activities were dramatically reduced following a conformational change in MRS when cognate tRNAs (initiator and elongator tRNA^Met^) were expressed. Unlike LgBit-MRS-SmBit, the luciferase activities of LgBit-DRS-SmBit (**D**) and LgBit-KRS-SmBit (**E**) were not reduced significantly in the presence of their cognate tRNAs. (**F**) LgBit-MRS-SmBit did not show a noticeable change in luciferase activity following methionine starvation or supplementation in either the presence and absence of the ribosome inhibitor cycloheximide (CHX).

In the closed conformation of the above MRS model, both GST and WHEP domains face the tRNA binding site. We confirmed this spatial arrangement of MRS domains *in vivo* using NanoBiT. AIMP3 containing the nanoluciferase large subunit (LgBit) at its N-terminus (LgBit-AIMP3) was prepared, and luciferase activity was measured with co-expression of the nanoluciferase small subunit (SmBit) at the N- or C-termini of MRS (SmBit-MRS and MRS-SmBit, respectively) in CHO-Ki cells. Since we already know the relative position of the MRS N-terminus relative to AIMP3 from the structure of the MRS_GST_-AIMP3 complex, the C-terminal position of MRS was estimated by comparison of the luciferase activity of the MRS-SmBit:AIMP3 pair with that of the SmBit-MRS:AIMP3 pair (Figure 10A). Background luciferase activity was measured first with cells expressing AIMP3 containing LgBit, and it was barely increased when SmBit was introduced by another AIMP3. This implies that two AIMP3 molecules do not form a homodimer and are not located close to each other in MSC. However, co-expression of SmBit-MRS increased luciferase activity dramatically (>120-fold), consistent with the formation of a heterodimeric complex between MRS_GST_ and AIMP3. Co-expression was then performed with MRS-SmBit, and luciferase activity was comparable with that measured when SmBit was expressed at the N-terminus of MRS. The C-terminus of MRS therefore appears to be located close to AIMP3, like the N-terminus of MRS, and this was indeed confirmed using AIMP3 containing LgBit at its C-terminus (AIMP3-LgBit). Co-expression of MRS containing SmBit increased luciferase activity dramatically. The activities of luciferases with the subunit at the N- and C-termini of MRS were similar to each other; luciferase activity with the nanoluciferase subunit at the C-terminus of AIMP3 was similar to that with the nanoluciferase subunit at the N-terminus of AIMP3. This may be because AIMP3 is a small, compact domain in which the N- and C-termini are not far from each other. The C-terminus of MRS is therefore close to both ends of AIMP3, as well as the N-terminus of MRS, in the MRS_GST_-AIMP3 complex.

To monitor the transition of MRS from closed to open conformations, we generated an MRS construct containing the large subunit at the N-terminus and the small subunit at the C-terminus (LgBit-MRS-SmBit). Since the N- and C-terminal ends of MRS are close to each other in the closed conformation, LgBit-MRS-SmBit should exhibit luciferase activity in the absence of tRNA. However, in the presence of tRNA, binding of tRNA to MRS should convert MRS to an open conformation. Interaction with tRNA will induce MRS to release the linker peptide from the ABD, and the N-terminal GST domain will be distant from the C-terminal end, resulting in loss of luciferase activity. To increase the chance of the conformational change taking place, we increased the expression of the tRNA^Met^ gene relative to expression of the gene encoding LgBit-MRS-SmBit, and luciferase activity decreased with increasing tRNA expression (Figure 10B).

To confirm that the decrease in luciferase activity was due to the binding of tRNA^Met^, we measured the activity of LgBit-MRS-SmBit following overexpression of various tRNA species. Compared with controls without tRNA overexpression, when cognate tRNA was overexpressed, luciferase activity was decreased significantly (Figure 10C). Specifically, >70% of activity was lost when initiator tRNA^Met^ was overexpressed, and only 20% of activity remained in the presence of elongator tRNA^Met^. Expression of noncognate tRNA also decreased luciferase activity, but it was not as effective as cognate tRNAs. To explore whether this decrease in luciferase activity is due to MRS-specific conformational changes induced by its cognate tRNA, we generated LgBit-DRS-SmBit and LgBit-KRS-SmBit and monitored their luciferase activities. As expected, tRNA^Asp^ and tRNA^Lys^ did not show dramatic changes in the luciferase activities of LgBit-DRS-SmBit and LgBit-KRS-SmBit, respectively (Figure 10D and E).

Using LgBit-MRS-SmBit, the effect of methionine on the MRS conformational change was monitored by starvation and supplementation of methionine (Figure 10F). Luciferase activity was not affected much under either condition. As expected with similar methionine activation activities of MRS with and without the N-terminal appendage, methionine does not enhance the transition of MRS to its open conformation. We also monitored the conformational change of MRS in the presence of the ribosome inhibitor cycloheximide (Figure 10F). Under methionine depleted and enriched conditions, MRS displayed similar luciferase activities in the presence and absence of cycloheximide.

## DISCUSSION

Human MRS has additional polypeptides at both the N- and C-termini of the conserved catalytic main body. The N-terminal peptide consists of a GST domain and a linker peptide of 73 residues between the GST domain and catalytic domain, while a linker region of 20 amino acids attaches a WHEP domain at the C-terminal end. Poor electron density in crystal structures indicates that both linker peptides have a degree of flexibility. Proteolysis of MRS in the MSC by trypsin generates a fully-functional truncated form that is released from the MSC (33). This 77 kDa protein lacks the N-terminal GST domain (214 amino acid residues) (20), indicating that the N-terminal linker region houses the protease cleavage site, which must be exposed to solvent for proteolysis to take place. The size (96 kDa) of the other truncated MRS protein resulting from digestion with trypsin, which remains associated with the MSC (33), also suggests that the C-terminal peptide linking the WHEP domain is susceptible to proteolysis.

The N-terminal linker in human MRS can be divided into three sections: an N-terminal peptide enveloping the GST domain, a C-terminal peptide attaching the catalytic main body, and a flexible region between the two peptides. Numerous glutamate residues in the flexible region may facilitate electrostatic interactions with the ABD rather than negatively-charged tRNA. In a full-length model of MRS, the flexible region envelops the tRNA binding site on the ABD of MRS, thereby blocking interaction with tRNA. This closed conformation of MRS is a tightly bound, compact part of the MSC. Binding of tRNA accompanies the release of the glutamate-rich linker peptide from the tRNA binding site, which allows movement of the GST domain away from the catalytic main body. In this open conformation, the linker peptide can be extended, and the GST domain is separate from the catalytic main body of MRS. Since MRS attaches to the MSC through interaction with AIMP3 via its N-terminal GST domain, this extended form of MRS contributes to an overall expansion of the MSC.

Herein, we propose two different conformations of MRS that are dependent on the position of the N-terminal flexible linker peptide. The first is a compact form in which the GST domain is located close to the catalytic domain, and the second is an open form in which the GST domain is apart from the catalytic main body. A more extended linker peptide can be envisaged. Even without the first helix of MRS_221–834_ in the N-terminal linker peptide attached to the ABD, MRS is still catalytically active (37), and the GST domain lacking helix α9 is sufficient for MRS to bind AIMP3 for integration into the MSC (6). This extension would allow spatial freedom for MRS to facilitate interaction with tRNAs and other binding partners such as kinases. Flexibility through the linker peptides is implicated in the non-canonical functions of MRS. Phosphorylation of human MRS by ERK occurs at S209 (23), located in the peptide region enveloping the GST domain of MRS, and this may affect the positions of the N-terminal linker peptide and the GST domain. Repositioning of MRS domains may expose AIMP3, which is also phosphorylated by ERK, and this allows its translocation to the nucleus (40). Phosphorylation would be facilitated when the MRS catalytic domain is separated from AIMP3. Other MRS phosphorylation sites have also been reported. Phosphorylation at S662 by GCN2 induces the release of AIMP3 from the MSC, and its subsequent translocation into the nucleus, where it functions as a tumor suppressor (22). S662, located at the end of helix α11b in the ABD, contacts a long peptide between helices α15 and α16 that envelops the ABD. This long peptide is positioned beneath the C-terminal section of the N-terminal linker peptide between the GST and catalytic domains. Phosphorylation at S662 would affect the local geometry of loops and helices, and the N-terminal linker may fully extend under these circumstances. The N-terminal GST domain of MRS plays a structural role in attaching to the MSC, while the linker plays a regulatory role through conformational changes of MRS.

Many eukaryotic MRS proteins have a GST domain at their N-terminus, and the GST domains of mammalian MRS proteins are connected to their catalytic Rossmann fold domains through a long linker peptide. The GST domain participates in the MSC via interaction with AIMP3, and the linker may ensure an appropriate spatial distance between the MSC and MRS. Yeast MRS, which also has a GST domain at its N-terminus, forms a primitive MSC with ARC1P and glutamyl-tRNA synthetase (ERS). The GST domain plays a role in interacting with the N-terminal GST-C-like domain of ARC1P (41), and yeast MRS in the MRS-Arc1p-EPRS complex displays increased charging efficiency (42).

The linker between the GST domain and the catalytic core in yeast MRS is relatively short compared with its counterpart in human MRS, and the yeast primitive MSC is smaller than the human MSC. The apicomplexan MSC is assembled from five components including MRS (2), which has an N-terminal GST domain linked through a short peptide. The architecture of MRS with a long linker is conserved in organisms from insects to mammals, all of which are similar in complexity to the human MSC, while MSCs in other organisms are simpler (43,44). The long linker between GST and catalytic domains may be implicated in the large size of the MSC assembly and/or the catalytic function of MRS, since the charging efficiency of MRS is important in the huge MSC.

Many MSC component proteins have flexible linkers between their structured domains (45). AIMP1 and AIMP2, both of which contain a heptad repeat forming a leucine zipper, possess EMAPII and GST domains, respectively (5). These domains are connected to the leucine zipper by long linker peptides. EPRS is a multi-domain protein consisting of four segments (GST, ERS, three WHEP domains, and PRS) from its N- to C-terminus. Each segment is joined to neighboring segments by a long linker peptide (24). Thus, MSC component proteins are likely to be conformationally dynamic and highly flexible. The three-dimensional structure of the whole MSC was investigated, and EM analysis revealed a globular assembly approximately 100–160 × 190 Å in size (7), while SAXS analysis indicated a more elongated form (8) ∼310 × 520 Å. These structural images are snapshots of the dynamic conformations adopted by the MSC, and may represent the most abundant shapes and sizes present under specific experimental conditions. The molecular weight of the MSC is ∼1.5 MDa, hence its volume can be estimated to be 1.85×10^6^ Å^3^ based on the specific volume of a protein (∼0.74 cm^3^/g). When we image the packing of component proteins in MSC as crystallographic packing in solution, the volume of MSC including solvent molecules in the void space between components can be calculated to be 3.75 × 10^6^ Å^3^ by applying a Matthew's coefficient of 2.5 per Dalton. This represents a cubic volume with a length of 150 Å. By comparison, approximate volumes of hexahedrons containing the MSC measured by EM and SAXS are 3.0 × 10^6^ Å^3^ and 5.0 × 10^6^ Å^3^, respectively. These volumes indicate compact and expanded forms of MSC, respectively.

The MSC may adopt both compact and extended conformations due to dynamic motions of MSC components, as shown for MRS. In a living cell, the MSC must be dynamic for tRNA charging and interaction with other cellular components. Long linker peptides between compact structural domains allow conformational changes that are essential for function, and dynamic motions in large, complex protein assemblies such as MSCs are particularly important.

## DATA AVAILABILITY

Atomic coordinates and structure factors for the reported crystal structures (MRS_221–834_ and the MRS_1–224_–AIMP3 complex) have been deposited with the Protein Data bank under accession numbers 5GL7 and 4BVY, respectively.
